# Evaluation of psychometric properties of the Dental Anxiety Inventory (DAI‐36) questionnaire using iterative hybrid ordinal logistic: Differential item functioning (DIF)

**DOI:** 10.1002/brb3.3129

**Published:** 2023-07-17

**Authors:** Narges Roustaei, Elahe Allahyari

**Affiliations:** ^1^ Department of Epidemiology and Biostatistics, School of Health and Nutrition Sciences Yasuj University of Medical Sciences Yasuj Iran; ^2^ Department of Epidemiology and Biostatistics, School of Health, Social Determinants of Health Research Center Birjand University of Medical Sciences Birjand Iran

**Keywords:** dental anxiety, differential item functioning, psychometric properties

## Abstract

**Objectives:**

Dental Anxiety Inventory (DAI‐36) questionnaire is an instrument for assessing dental anxiety. The different perceptions of the questionnaire items in the individual at the same level of underlying dental anxiety may lead to different reported dental anxieties. This study aims to determine the differential item functioning (DIF) of the DAI‐36 measure items.

**Methods:**

The DAI‐36 was completed by 950 participants. An iterative hybrid ordinal logistic regression model was used to detect DIF across gender, education, and age groups. DIF analysis was done by lordif package in R3.1.3 software.

**Results:**

The chi‐square statistics declared 7, 7, and 4 nonuniform DIF items, and 2, 5, and 4 uniform DIF items across gender, education, and age groups, respectively. Δ*R*2 was always lower than 0.07 in all uniform and nonuniform DIF items. However, Δ*β*1 revealed significant uniform DIF in items 1 and 8 across gender (Δ*β*1(item 1) = 0.0137, Δ*β*1(item 8) = 0.0124) and items 22 and 27 across age categories (Δ*β*1(item 22) = 0.0110, Δ*β*1(item 27) = 0.0136).

**Conclusions:**

DIF items had no large magnitude or cancel out each other, so statements phrased in the DAI‐36 questionnaire have equivalent meaning across participants, regardless of their gender, education, and age groups.

## Graphical abstract

The different perceptions of the items of the DAI‐36 questionnaire in the individual at the same level of underlying dental anxiety may lead to different reported dental anxiety. This study aims to determine whether people of different gender, age, and education levels respond differently to the items in the Persian version of the DAI‐36 measure using the approach of differential item functioning (DIF). This study showed, participants' perception of the questionnaire's items was the same for both levels of gender, age, and, education. Therefore, the validation of the questionnaire was not affected by uniform DIF items across these factors.

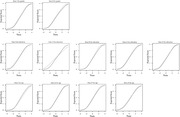

## 1 INTRODUCTION

Dental anxiety is an aversive emotional state of fear and an important issue in children and even adults (Bradt & Teague, 2018; Caltabiano et al., 2018). Different studies reported that approximately 50% of children and 10%–20% of adults experienced dental anxiety in different countries (Bradt & Teague, 2018; Campos et al., 2013; Malvania & Ajithkrishnan, 2011; Vlad et al., 2020). Dental anxiety leads to a lack of patient management, extended duration of dental appointments, increased dental care costs, and avoidance of dental treatment (Abanto et al., 2017; Bradt & Teague, 2018; Caltabiano et al., 2018; Malvania & Ajithkrishnan, 2011). Avoidance of dental treatment causes poor oral health, negatively impacting the oral health‐related quality of life (Caltabiano et al., 2018; Grisolia et al., 2020).

According to the literature, different factors were associated with dental anxiety. Gender and age were the significant factors in dental anxiety (Caltabiano et al., 2018; Campos et al., 2013; Malvania & Ajithkrishnan, 2011; Yuan et al., 2008). Women had more dental anxiety than men (Chavez et al., 2013; Malvania & Ajithkrishnan, 2011; Yuan et al., 2008). Moreover, with increasing age, dental anxiety decreased (Caltabiano et al., 2018; Yuan et al., 2008). The level of education was not a significant factor in dental anxiety (Campos et al., 2013; Chavez et al., 2013; Malvania & Ajithkrishnan, 2011). The difference in the dental anxiety between groups (e.g., gender and age) may be reflected in the perception of individuals of the items of the questionnaire at the same level of underlying dental anxiety. In other words, due to the invalidity of the measurement tool in one or more groups, the observed difference between the groups may be artificial (Rouquette et al., 2019). So, it is vital to use valid psychological science properties tools to gather data with prime quality (Campos et al., 2013).

There are different psychometric properties measuring tools for assessing dental anxiety such as the Dental Anxiety Inventory (DAI), the Dental Anxiety Scale (DAS), the Dental Fear Survey, the Modified Dental Anxiety Scale , the State‐Trait Anxiety Inventory Scale for children, Abeer Children Dental Anxiety Scale, and the others (Caltabiano et al., 2018; Campos et al., 2013; Ikeda & Ayuse, 2013; Vlad et al., 2020).

The DAI questionnaire is one of the more comprehensive tools that consider the range of dental anxiety and the multifaceted nature of dental anxiety (Aartman, 1998; Ikeda & Ayuse, 2013). Traditional validation procedures, such as criterion, structure, convergent, and discriminant, have been applied to this questionnaire (Aartman, 1998; Ikeda & Ayuse, 2013; Stouthard et al., 1995).

Differential item functioning (DIF) is an alternative and complementary validation approach to exploring the psychometric properties of the tools. When different groups of test subjects with the same general ability, or the same situation based on an appropriate criterion, have systematically different responses to a particular item average, the used instrument will not be a suitable tool for comparison. Therefore, DIF analysis confirms the correctness of the group comparison by examining this aspect of validity.

This study aimed at assessing dental anxiety in a generally healthy population and examine DIF across gender, age, and education level for the DAI‐36 questionnaire using the hybrid ordinal logistic regression (OLR/IRT) model.

## 2 METHOD

### 2.1 Subjects

Before data collection, the code of ethics (Ir.bums.REC.1398.296) was obtained from Birjand University of Medical Sciences Ethics Committee. Then, we divided each of the cities of Birjand, Mashhad, and Shiraz into four geographical districts (north, east, west, and south). Moreover, 40 households were randomly selected in each district from the list of telephone numbers in telecommunications. Then, two family members (one male and one female) who lived in each of the 40 randomly selected households were asked to complete the DAI‐36 questionnaire and the initial information checklist (including age, educational level, and gender). Only individuals who had more than 18 years old and did not have a history of mental illness were interred in the study. Individuals received explanations regarding the study, and people who did not want to participate in the study were replaced. Then, participants were asked to sign informed consent forms. The questionnaire for illiterate subjects was completed through interviews by the interviewer. Finally, 10 incomplete questionnaires were excluded before data analysis, and this study used information from 950 remaining participants.

### 2.2 Instrument

The DAI questionnaire included 36 items on a 5‐point scale that people rate their dental anxiety from 1 (totally untrue) to 5 (completely true) (Stouthard et al., 1993). A high correlation between the DAI and Corah's DAS supported convergent validity. The original DAI version conjointly had discriminant validity, thanks to a nonsignificant correlation with variables like sociableness and little positive correlations with scales for mental disorder, anxiety, and fear. Marlies’ study conjointly supported the construct validity of the DAI. The Persian version had additionally high internal consistency (Cronbach's alpha = .94 and *r*_split‐half_ = .95) (Yousefi & Piri, 2017).

### 2.3 Data analysis

One of the common methods for determining the fairness of test components through demographic subgroups can be referred to the DIF (Lambert et al., 2018). When underlying true ability was the same for subjects of separate subgroups, but they have a different probability of giving a certain response to an item. That item cannot measure abilities for members of separate subgroups in the same way because of different interpretations. Therefore, such items are not able to be used for valid and fair comparisons. DIF analysis determines such items.

DIF is categorized into two types: uniform and nonuniform. If the reaction of one group is always higher or lower than other groups at all levels of matching variables, then it could be located in uniform; otherwise, it is nonuniform DIF. The subgroups cannot be compared in large uniform DIF items.

The OLR determined uniform and nonuniform DIF by comparing Models 1 and 2 and Models 2 and 3, respectively (Zumbo, 1999):

$$\mathrm{logit}[P(Y\leq k|g,\theta )]=\beta_{0k}+\beta_{1}\theta \;k=0,1,\text{…},m-1\;\left(\mathrm{Model}1\right)$$

$$\mathrm{logit}\left[P\left(Y\leq k|g,\theta \right)\right]=\beta_{0k}+\beta_{1}\theta +\beta_{2}g\;k=0,1,\text{…},m-1\;\left(\mathrm{Model}2\right)$$

$$\mathrm{logit}\left[P\left(Y\leq k|g,\theta \right)\right]=\beta_{0k}+\beta_{1}\theta +\beta_{2}g+\beta_{3}\theta g\;k=0,1,\text{…},m-1\;\left(\mathrm{Model}3\right)$$

In these formulas, *m*, *θ*, and *g* were assumed to be the number of domains, ability score, and grouping variable, respectively. To adjust the effect of the biased item on DIF detection, an iterative hybrid OLR incorporates the Rasch trait score rather than the sum of score ability and uses an iterative procedure to detect DIF items, as described previously (Choi et al., 2011).

In large sample size studies, the statistically significant *χ* ^2^ test without using parallel DIF effect size measures might be misguiding (Cohen, 1977). So, we used Crane van Belle and Larson's criterion (CVBL) as a uniform DIF effect size measure and McFadden pseudo‐*R*‐square (Δ*R^2^*) as both uniform and nonuniform DIF effect size measures (Crane et al., 2004; Holland & Thayer, 1988). McFadden pseudo‐*R* ^2^ higher than 0.070 and CVBL higher than 0.01 are considered large DIF measures in the present article. So we focused on declaring the DIF items of the DAI‐36 by the lordif package in R3.1.3 software (Choi et al., 2011). Finally, comparing between groups was analyzed by *T*‐test and Mann–Whitney *U*‐test with and without DIF items.

## 3 RESULTS

In this study, 950 respondents (492 males and 458 females) answered the DAI questionnaire. The mean (SD) ages of males and females were 33.1 (11.63) and 28.74 (9.28) years, respectively (54.9% lower than 30 years old, 45.1% equal or more than 30 years old). Most of the participants were nongovernment employees (30.3% students, 28% government employees, and 41.7% nongovernment employees), lived in the city (80%), and had no academic degree (54.5%). In Table 1, the confirmatory factor analysis (CFA) indices were reported for the total DAI‐36 score and each gender, education, and age category separately. As Table 1 clearly shows, Cronbach's alpha, coefficient omega, and the standardized root mean squared residuals were in an acceptable range. But all other CFA indices except total root mean squared error of approximate were out of recommended range by Hu and Bentler (Brown, 2014; Hu & Bentler, 1999).

**TABLE 1:** The results of validity and reliability in each demographic factor category separately.

		Alpha^a^	Omega^b^	RMSEA^c^	SRMR^d^	CFI^e^	TLI^f^
Gender	Male	.97^g^	.97^g^	.066	.052^g^	.876	.868
	Female	.97^g^	.97^g^	.068	.051^g^	.871	0.863
Education	Non‐academic	.96^g^	.98^g^	.077	.058^g^	.830	.820
	Academic	.97^g^	.98^g^	.073	.056^g^	.859	.851
Age	<30	.97^g^	.98^g^	.092	.070^g^	.786	.773
	≥30	.97^g^	.98^g^	.086	.063^g^	.812	.801
Total		.97^g^	.97^g^	.060^g^	.039^g^	.899	.893

^a^ Cronbach's alpha coefficient.

^b^ Guttman's Lambda 6 reliability.

^c^ Root mean squared error of approximate (RMSEA).

^d^ Standardized root mean squared residual (SRMR).

^e^ Comparative fit index (CFI).

^f^ Tucker–Lewis index (TLI).

^g^ Model fit indices acceptable range recommended by Hu and Bentler.

### 3.1 DIF analysis

DIF analysis is provided in Table 2. The chi‐square statistics detected nonuniform DIF for 7, 7, and 4 questions of the questionnaire across gender, educational l, and age categories, respectively. But, Δ*R* ^2^ was always lower than 0.008.

**TABLE 2:** The results of the hybrid OLR/IRT differential item functioning (DIF) analysis on the Dental Anxiety Inventory (DAI).

	Nonuniform		Uniform		
	*χ* ^2^(*P*)	Δ*R*_2_	*χ* ^2^(*P*)	Δ*R*_1_	∆*β*_1_
Gender					
1. I become nervous when the dentist invites me to sit down in the chair	.351 (.5534)	.0001	8.239 (.0041^a^)	.0029	.0137^a^
3. When I'm on my way to the dentist and thinking about the anesthetic, I would rather go back	6.784 (.0092^a^)	.0025	2.024 (.1548)	.0007	.0073
8. I already feel uncomfortable at home when I think that the dentist will make a remark about my teeth	.394 (.5303)	.0001	6.224 (.0126^a^)	.0023	.0124^a^
12. When I think of the moment when the dentist blows air into a cavity, I would like to cancel the appointment	6.582 (.0103^a^)	.0024	.4335 (.5103)	.0002	.0046
15. On my way to the dentist, I feel nervous when I know my teeth will be scaled	7.247 (.0071^a^)	.0027	.319 (.5720)	.0001	.0043
22. In the waiting room, I sweat or freeze when I think of sitting down in the dentist's chair	5.017 (.0251^a^)	.0018	1.01 (.3152)	.0004	.0052
27. On my way to the dentist, I get anxious at the thought that she/he will have to drill	6.989 (.0082^a^)	.0025	.098 (.7537)	.0000	.0021
34. When I'm waiting for the dentist's assistant to call me in, I try to think of something else	4.762 (.0291^a^)	.0018	2.701 (.1003)	.0010	.0127
36. I sleep badly the night before I have to have a tooth extracted	15.138 (.0001^a^)	.0054	1.863 (.1723)	.0007	.0120
Education					
3. When I'm on my way to the dentist and thinking about the anesthetic, I would rather go back	5.238 (.0221^a^)	.0019	.023 (.8784)	.0000	.0004
8. I already feel uncomfortable at home when I think that the dentist will make a remark about my teeth	5.360 (.0206^a^)	.0020	1.064 (.3023)	.0004	.0014
9. When the dentist is about to give me an anesthetic, I cling to the arms of the chair	.102 (.7500)	.0000	5.577 (.0182^a^)	.0020	.0078
10. I become afraid in the waiting room when I hear sounds coming from the dentist's surgery	7.0102 (.0077^a^)	.0025	1.789 (.1810)	.0006	.0016
14. I want to walk out of the waiting room the moment I think the dentist will not explain what she/he is going to do in my mouth	6.371 (.0116^a^)	.0023	.202 (.6531)	.0001	.0011
15. On my way to the dentist, I feel nervous when I know my teeth will be scaled	12.532 (.0004^a^)	.0048	6.013 (.0142^a^)	.0023	.0076
20. Before going to the dentist, I get palpitations when I think of how the dentist will be displeased at my teeth	2.188 (.1391)	.0008	5.586 (.0181^a^)	.0020	.0097
21. As soon as the dentist gets his/her needle ready for the anesthetic, I shut my eyes tight	.494 (.4820)	.0002	4.774 (.0289^a^)	.0017	.0083
34. When I'm waiting for the dentist's assistant to call me in, I try to think of something else	1.371 (.2417)	.0005	5.636 (.0176^a^)	.0021	.0019
35. On my way to the dentist, the idea of being in the chair already makes me nervous	4.750 (.0293^a^)	.0018	.397 (.5285)	.0001	.0018
36. I sleep badly the night before I have to have a tooth extracted	5.511 (.0189^a^)	.0019	1.054 (.3047)	.0004	.0020
Age					
2. I need to go to the toilet more often when I sit in the waiting room thinking that the dentist will say my teeth look bad	4.923 (.0265^a^)	.0018	13.070 (.0003^a^)	.0048	.0042
11. On my way to the dentist, I sweat or freeze at the thought that the dentist will say I brush my teeth badly	15.138 (.0001^a^)	.0071	.670 (.4130)	.0002	.0040
22. In the waiting room, I sweat or freeze when I think of sitting down in the dentist's chair	.282 (.5952)	.0001	5.447 (.0196^a^)	.0020	.0110^a^
26. In the waiting room, I feel nervous at the thought that the dentist will say my teeth are badly brushed	5.596 (.0180^a^)	.0020	.735 (.3913)	.0003	.0042
27. On my way to the dentist, I get anxious at the thought that she/he will have to drill	.053 (.8187)	.0000	7.033 (.0080^a^)	.0025	.0136^a^
29. When I am sitting in the dentist's chair not knowing what is going on in my mouth, I break into a cold sweat	15.138 (.0001^a^)	.0063	.065 (.7993)	.0000	.0006
36. I sleep badly the night before I have to have a tooth extracted	2.193 (.1386)	.0008	4.168 (.0412^a^)	.0015	.0083

*Note*: *P*: *p*‐value of chi‐square statistic; *χ* ^2^: the value of the difference in −2 log likelihood of models with and without group variable *G* for uniform DIF and models with and without interaction *θG* for nonuniform DIF; Δ*β*1: Crane van Belle and Larson criterion or |[*β*_1_ (Model_without_ *_G_*) − *β*_1_ (Model_with_ *_G_*)]/*β*_1_ (Model_without_ *_G_*)|. Δ*R*_1_ = 1 − ln[*L*(Model_with_ *_G_*)]/ln[*L*(Model_without_ *_G_*)]; Δ*R*_2_ = 1 − ln[*L*(Model_with_ *_θG_*)]/ln[*L*(Model_without_ *_θG_*)].

^a^ Items showing uniform or nonuniform DIF.

The chi‐square statistics also reported significant uniform DIF in items 1 and 8 of the questionnaire across gender categories, items 9, 15, 20, 21, and 34 across educational levels, and items 2, 22, 27, and 36 across age categories. However, none of Δ*R*_1_ was large (Δ*R*_1_ ≤ 0.48). Δ*β*_1_ was significant in uniform DIF questions across gender (Δ*β*_1_(item 1) = 0.0137, Δ*β*_1_(item 8) = 0.0124) and questions 22 and 27 across age categories (Δ*β*_1_(item 22) = 0.0110, Δ*β*_1_(item 27) = 0.0136).

**FIGURE 1:**
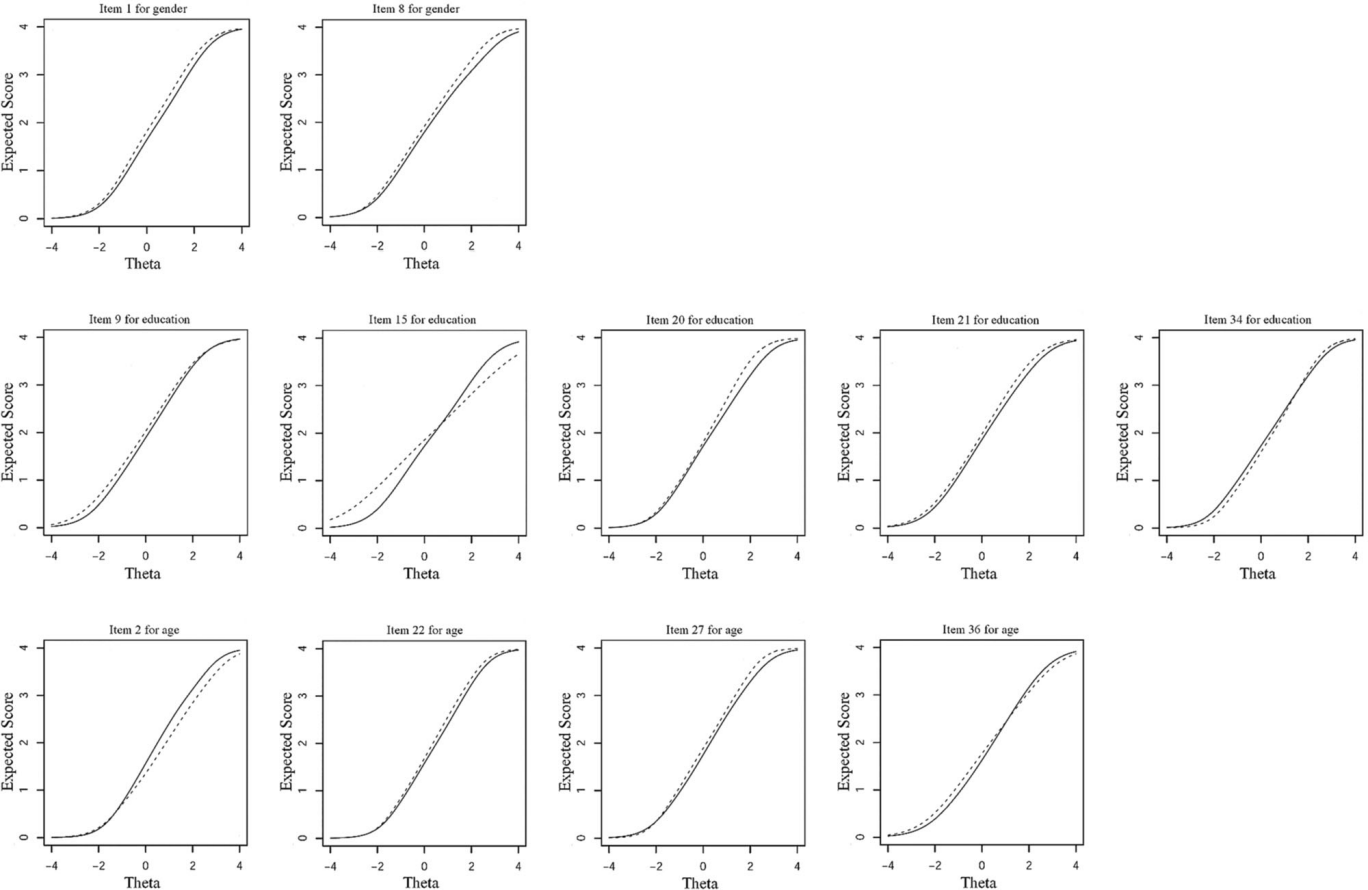
Test characteristic curves of differential item functioning (DIF) items for gender (male [solid line], female [dashed line]), education (nonacademic [solid line], academic [dashed line]), and age (<30 [solid line], ≥30 [dashed line]) groups according to the hybrid OLR/IRT.

Figure 1 shows the additive or cancel‐out effect of uniform DIF items in gender, educational level, and age variables (Figure 1). Items 1 and 8 of the questionnaire had uniform DIF across gender groups. These items had small Δ*R*_1_; therefore, there was no important effect on the valid interpretation of gender group differences (Table 2). Items 34 and 9 of the questionnaire had uniform DIF across educational levels and cancel out each other, but items 15, 20, and 21 had uniform DIF and go in one direction. Δ*R*_1_ and Δβ_1_ had a small magnitude; therefore, these items cannot change group comparison between subjects with and without an academic degree.

In the third row of Figure 1, only nonsignificant item 2 was in the opposite direction of items 22, 27, and 36, but additive effects of 22, 27, and 36 were not important in assessing age groups difference because of the very small Δ*R*_1_ (range 0.0015–0.0025).

To assess whether uniform DIF questions affected the valid interpretation of group differences, the mean score of DAI‐36 was compared between levels of gender, education, and age considering with and without uniform DIF questions in Table 3.

**TABLE 3:** The results of comparing group differences with and without uniform differential item functioning (DIF) items.

		Total score		Total score corrected for DIF	
		Mean (SD)	*p*‐Value^b^	Mean (SD)	*p*‐Value^b^
Gender^a^	Male	2.765 (.745)	.269	2.759 (.745)	.318
	Female	2.712 (.725)		2.711 (.725)	
Education^c^	Non‐academic	2.700 (.719)	.070^d^	2.684 (.726)	.108
	Academic	2.787 (.753)		2.770 (.756)	
Age^e^	<30	2.603 (.707)	**<.001**	2.609 (.715)	**<.001**
	≥30	2.906 (.736)		2.913 (.730)	

^a^ Total score after removing uniform DIF items 1 and 8.

^b^ *p*‐Value of Mann–Whitney *U*‐test.

^c^ Total score after removing uniform DIF items 9, 15, 20, 21, and 34.

^d^ *p*‐Value of independent samples *T*‐test.

^e^ Total score after removing uniform DIF items 2, 22, 27, and 36.

The DAI‐36 score between males and females, and between academics and nonacademics, had no significant difference in the presence of items with and without uniform DIF. Regarding age groups, older people had a higher score of the DAI‐36 than younger ones in the presence of items with and without uniform DIF (*p*‐value ≤ .001). These findings approved that the uniform DIF items had no significant effect on the comparison of different levels of gender, education, and age as well as a result of Figure 1 and Table 2.

## 4 DISCUSSION

This study aimed to evaluate one of the psychometric properties of the DAI‐36 questionnaire by hybrid OLR/IRT model and detecting DIF items. Two significant uniform‐DIF items that were detected across genders had no significant measure. Therefore, women did not report their dental anxiety more than men at the same level of dental anxiety. Moreover, the validation of the questionnaire was not affected by uniform DIF items across gender.

The total score of dental anxiety had not a statistically significant difference between women and men in some studies like our findings (Caltabiano et al., 2018; Campos et al., 2013; Kanegane et al., 2009), whereas women had more dental anxiety than men in some previous research (Chavez et al., 2013; Ikeda & Ayuse, 2013; Malvania & Ajithkrishnan, 2011; Prihastari et al., 2020; Tunc et al., 2005; Yuan et al., 2008). In several cultures, women more than men represented physiological conditions involving anxiety, worry, and fear (Arslan et al., 2011; Ikeda & Ayuse, 2013). This could be explained by the fact that although women are more sensitive about oral health caries, tooth loss was slightly more prevalent among them (Bonsall, 2014), and they had phobia before dental treatment of local anesthetic injection and tooth drills, also they reported lower pain thresholds (Caltabiano et al., 2018).

Although the academic groups overestimate dental anxiety more than the nonacademic, these DIF magnitudes were small. Therefore, perceiving the meaning of these items between academic and nonacademic groups was consistent. Patients’ perception of the questionnaire's items was the same for both educational levels. Therefore, the validation of the questionnaire was not affected by these uniform DIF items across educational levels.

The total score of the DAI‐36 questionnaire was not a statistically significant difference between academic and nonacademic groups. This finding is following the previous research showing that educational level was not a significant factor in dental anxiety (Campos et al., 2013; Fayad et al., 2017; Kanegane et al., 2009; Malvania & Ajithkrishnan, 2011; Saatchi et al., 2015).

This study revealed that patients with age ≥30 compared to those aged <30 responded different to four items in the DAI‐36 questionnaire. However, the validation of the questionnaire was not affected by these items because of small DIF magnitude. Moreover, patients with age ≥30 years old experienced more dental anxiety than those <30 years old. Older patients were possibly concerned about many, and large dental problems also had been referred to a dentist with a bad dental experience commonly; therefore, they had more reported their dental anxiety than younger.

Similar studies showed that dental anxiety in patients, the 35–49 years, was the highest (Stabholz & Peretz, 1999) and in adults, 31–35 years, was high and decreased after 60 years old (Svensson et al., 2016). In a study, about 17% of adults reported exacerbated dental anxiety in the waiting room of a clinic (Chavez et al., 2013). However, in some previous studies, the younger age group had the highest dental anxiety scores than the older age group (Hägglin et al., 2000; Yuan et al., 2008). It might be decreased compatibility with increasing age, and older may have some disabilities so they more reported their anxiety.

### 4.1 Strengths and limitations

Finally, this is the first study to evaluate DIF items of the DAI‐36 questionnaire across gender, age, and level of education using the hybrid OLR/IRT model. So, other studies in different cultures can help us to ensure the good validity of these questions for different translations.

## 5 CONCLUSION

Our study revealed that some items in the DAI‐36 questionnaire did not function in a similar way across women and men and different levels of age and education. However, patients’ perception of the questionnaire's items was the same for both levels of gender, age, and education. Therefore, the validation of the questionnaire was not affected by uniform‐DIF items across these factors.

The management of the patient's dental anxiety is important because of avoidance of dental treatment; therefore, dental anxiety may cause poor oral health and oral health‐related quality of life. Moreover, dentists should be cautious about ensuring patient comfort for dental procedures, especially in older patients.

### PEER REVIEW

The peer review history for this article is available at https://publons.com/publon/10.1002/brb3.3129.

## Data Availability

The datasets used and/or analyzed during the current study are available from the corresponding author on reasonable request.
